# Research on the measurement and characteristics of museum visitors’ emotions under digital technology environment

**DOI:** 10.3389/fnhum.2023.1251241

**Published:** 2023-09-04

**Authors:** Ting Zhang, Zipeng Qi, Weiwei Guan, Cheng Zhang, Dingli Jin

**Affiliations:** ^1^Economics and Management School, Wuhan University, Wuhan, Hubei, China; ^2^Ningbo National Institute Insurance Development (NIID), Wuhan University, Ningbo, China; ^3^China Yangtze Power Co., Ltd, Wuhan, Hubei, China

**Keywords:** museum tourism, visitor emotional measurement, psychology, PAD emotion model, EEG signals

## Abstract

What kind of emotional experience does the application of digital technology in museums create for museum visitors? Can it be measured accurately and in real-time? What are its characteristics? This paper utilizes EEG signals and the PAD emotional model as research methods to conduct real-time digital measurement of visitors’ emotional experiences at Tianyi Pavilion Museum in Ningbo City, focusing on their physiological and psychological reactions.The results show that: (1) In a quasi-experimental environment, linear SVM, polynomial kernel SVM, and Gaussian kernel SVM can all accurately classify the emotional tendencies of museum visitors with success rate of over 72%. (2) In a quasi-experimental environment, it is feasible and reliable to measure the immediate digital emotional experiences of visitors using EEG signals and the PAD emotion model. Based on this, we can summarize the characteristics of emotional tendencies among different demographic groups of museum visitors.

## 1. Introduction

With the advancement of technologies such as 5G, AR, VR, and artificial intelligence, digital tools like Unreal Engine, digital twins, and holographic imaging are increasingly being utilized in museum tourism. Research indicates that the extensive implementation of digital technology in museum tourism can heighten visitors’ emotional experiences (Ning et al., 2022). Therefore, what types of emotional experiences do museum-goers encounter when utilizing digital technology applications? Can these encounters be instantly and accurately quantified?

The assessment of tourists’ affective responses has always been a focal point and challenge in the investigation of emotional experiences within tourism (Sun and Li, 2021; Zhao et al., 2023). After reviewing related studies, it can be identified that the existing research on calculating museum visitors’ emotions has several issues. Firstly, there is a lack of diversity in calculation methods as most studies rely on questionnaires and visitor comments (Zheng et al., 2022). Fewer studies directly measure visitors’ physiological responses, and even fewer combine both methods. Secondly, there is room for improvement in terms of instantaneity and accuracy. A few scholars have begun to focus on the immediate emotional experiences of museum visitors (Tu and Wang, 2023), utilizing offline field tracking research and participatory observation methods. However, there is still room for improvement in accurately and immediately measuring visitors’ emotions.

In fact, extensive research has been conducted on methods for measuring emotions in the field of psychology (Yin et al., 2023). Currently, the primary approaches utilized are physiological response (Lin et al., 2013). In the field of psychophysiological measurement methods, research on using EEG signals to measure emotions has reached a relatively mature stage. Numerous studies have demonstrated that detecting EEG signals can effectively identify emotions and offers advantages such as good identification performance and high accuracy (Chai et al., 2021; Wang C. et al., 2022). This method has been widely applied in fields such as education and healthcare; however, its application in the tourism industry remains limited. In terms of methods for measuring psychological responses, there are various options available, including the PrEmo emotion measurement method, AS emotional slider measurement method, PAD emotion measurement method, and semantic differential method (Xie et al., 2011; Chen et al., 2022). Among them, the PAD emotional model exhibits high applicability and has been extensively employed in domains such as emotional psychology, product design, and product satisfaction evaluation (Li and Sun, 2011). While some scholars in the tourism industry have attempted to employ this methodology for research purposes, its application within museum tourism remains limited.

Therefore, we approach the digital technology application environment from a visitor psychology perspective and utilize brainwave signals and the PAD emotional model to comprehensively measure museum visitors’ instant digital emotional experience based on their physiological and psychological responses. We enhance research on the physiological measurement of visitors’ emotional experiences, enhancing the accuracy and immediacy of emotional measurement, and summarizing the emotional tendencies of museum visitors in digital technology applications.

## 2. Position in the existing literature

### 2.1. Research on the measurement of visitor emotions in museums

Currently, research on the measurement of visitor emotions in museums mainly adopts survey observation and analysis of visitor comments. In terms of survey observation methods, Du and Li (2022) conducted offline field tracking surveys and participatory observation to study the spatial and temporal changes in the emotional experiences of domestic and foreign tourists in scenic areas (Du and Li, 2022). Zhang and Xu (2022), based on Straussd’s grounded theory, conducted semi-structured interviews and participatory observation to study the true emotions of museum visitors (Zhang and Xu, 2022). In terms of analysis of visitor comments, Zheng et al. (2022) achieved up to 95% performance in aspect-level sentiment classification based on content extraction and deep learning (Zheng et al., 2022).

However, the above studies are relatively traditional in terms of measurement methods, as they did not directly measure visitors’ physiological responses in real-time. They are primarily focused on qualitative analysis, and further research is needed to explore the immediacy, timeliness, and accuracy of visitor emotional experiences in museums. In fact, there are relatively abundant achievements in improving the immediacy, timeliness, and accuracy of emotional measurement from the perspective of psychological and physiological responses in psychology.

### 2.2. Regarding the measurement methods of psychological responses and emotions in psychology

Despite the various methods available in psychology for measuring emotional responses, the PAD three-dimensional model proposed by scholars such as Mehrabian has been widely accepted by researchers in the field of emotion research (Wang W. et al., 2022). The PAD emotional model offers a rapid and intuitive means of measuring emotions, with the added benefit of being applicable across diverse cultural contexts, thereby circumventing any cognitive disparities in adjective semantics. In comparison to alternative approaches, it boasts a broader range of potential applications and can be implemented in an array of different scenarios. Considering the differences between domestic and foreign samples, we used a modified simplified Chinese version of the PAD emotional scale developed by the Chinese Academy of Sciences. The internal consistency coefficients (Cronbach’s alpha) of this scale are 0.85, 0.58, and 0.72, indicating good reliability and construct validity (Li et al., 2008).

The Chinese version of the PAD emotional scale measures emotions from three aspects: pleasure, arousal, and dominance. Pleasure (abbreviated as “P” in the following text as well) refers to the positivity or negativity of a user’s emotional state;^1^ a positive P represents a positive emotional state, while a negative P represents a negative emotional state. Arousal (A) refers to the level of neural physiological activation and excitement of the user; a positive A indicates a higher level of excitement, while a negative A indicates a lower level of excitement. Dominance (D) refers to the strength of a user’s control over external situations or others. A positive D represents a dominant and proactive interaction, while a negative D represents a weaker control and being influenced by others.

The calculation steps of this model are as follows: first, based on the user’s scores on the PAD emotional scale, the P, A, and D values of the user’s emotions are calculated. Then, the emotional space distance $L_{n}$ is calculated (see formula 1). Finally, referring to the 14 basic emotions reference table (Table 1), the user’s emotional tendency^2^ is determined and classified as positive or negative (Jiang et al., 2021).


(1) $$L_{n}=\sqrt{{\left(P-p_{n}\right)}^{2}+{\left(A-a_{n}\right)}^{2}+{\left(D-d_{n}\right)}^{2}}\;\left(n=\left[1,14\right],n\in Z\right)$$

**Table 1:** Reference table of PAD values for 14 basic emotions.

Number	Emotional type	P	A	D
1	Joy	2.77	1.21	1.42
2	Optimism	2.48	1.05	1.75
3	Relaxation	2.19	−0.66	1.05
4	Surprise	1.72	1.71	0.22
5	Gentleness	1.57	−0.79	0.38
6	Dependence	0.39	−0.81	−1.48
7	Boredom	−0.53	−1.25	−0.84
8	Sadness	−0.89	0.17	−0.7
9	Fear	−0.93	1.3	−0.64
10	Anxiety	−0.95	0.32	1.02
11	Contempt	−1.58	0.32	−0.63
12	Disgust	−1.8	0.4	0.67
13	Resentment	−1.98	1.1	0.6
14	Hostility	−2.09	1.00	1.12

Some studies have empirically verified that the PAD emotional values and emotional tendencies in different fields such as product development, education, and marketing are consistent with the emotional experiences expressed by the experiment participants. However, the application of the PAD emotional model in museum tourism is currently limited, and further empirical research is needed to supplement the measurement of museum visitors’ emotions using this method.

### 2.3. Regarding the measurement methods of psychological and physiological emotional responses

The measurement techniques of psychological and physiological emotional responses are mainly based on the measurement of central nervous system activity in the brain or physiological responses associated with central nervous system activity (Wang Y. et al., 2022). Currently, emotion measurement research based on EEG signals is relatively mature. A large number of studies have shown that there is a certain correlation between EEG signals and emotions (Gao et al., 2022). Emotion recognition based on EEG signals has become a commonly used method and has been recognized by most scholars (Wang Z. et al., 2022). EEG signals are a type of electrophysiological signal that is usually obtained by placing electrodes on the scalp of the brain (Sun et al., 2021). According to their generation methods, they can be divided into spontaneous EEG and evoked EEG. Spontaneous EEG signals can reflect changes in the body’s own state and can be divided into five frequency bands: delta waves, theta waves, alpha waves, beta waves, and gamma waves. Evoked EEG refers to EEG signals generated under external stimulation, and the induction methods mainly include somatosensory evoked potentials and visual evoked potentials.

The steps of EEG emotion measurement mainly include the acquisition and preprocessing of EEG signals, feature extraction of EEG signals，emotion recognition and classification, analysis of emotion results. With the development of portable EEG devices and rapid advancements in signal processing methods, portable EEG devices produced by companies like Neurosky have effectively solved the problems of high cost, complex operation, and limited use in professional laboratories associated with traditional EEG devices (Sun et al., 2021). This provides technical support for the acquisition and processing of EEG signals in emotion measurement. Therefore, the key to using EEG signals for emotion measurement lies in EEG signal feature extraction and emotion recognition.

Regarding the feature extraction of EEG signals, signal spectral energy, power spectral density, and sample entropy are mainly used as EEG features for emotion recognition (Li et al., 2022). We mainly adopt signal spectral energy as the EEG feature for emotion recognition. Signal spectral energy mainly reflects the rhythmic characteristics of EEG signals through changes in signal amplitude and is often used as one of the physiological signal features to measure emotional changes. Previous studies have shown that energy values can significantly differentiate negative and positive emotions, making them suitable as EEG features for emotion recognition research (Teng et al., 2022).

In the research of emotion recognition based on EEG, some scholars mainly adopt machine learning algorithms for emotion recognition. Previous studies have shown that the support vector machine achieved an accuracy of 77.62, 78.96, and 77.60% in accurately identifying emotional valence, arousal, and dominance, respectively (Nawaz et al., 2020). Through literature review, it is found that SVM classifier is a fast and reliable linear classifier with good generalization capability and advantages in feature learning and numerical optimization. It can achieve more accurate EEG emotion classification for a second classification of emotions (Islam et al., 2021). The application of SVM in emotion classification is supported by a diverse range of inducing materials, encompassing not only the identification of emotions elicited by visual stimuli such as movies and video clips, but also those induced by olfactory cues. We also adopt this method for emotion measurement research.

Through comprehensive analysis, it can be inferred that in the realm of investigating measurement techniques for psychological and physiological responses to emotions, a majority of scholars have discovered that in experimental settings, machine learning algorithms can effectively recognize the experimenter’s emotions by analyzing EEG signals. However, there still room for research in the following aspects: (1) The experimental environment is strictly required, which is not conducive to application in actual environments. The signal acquisition environment is mostly concentrated in the experimental laboratory environment. Can still accurately identify experimenters’ emotions in the quasi-experimental environment, and it is expected to be further studied. (2) The experimental field is still expanding. At present, the research on emotion recognition of EEG emotion mainly focuses on the medical field, and the use of machine learning algorithms in museum tourism field is relatively small, and it is expected to be further supplemented.

Therefore, regarding the research on emotion measurement of museum tourists, most scholars have focused on measuring tourists’ emotional experiences from the perspective of their psychology. However, there are still three shortcomings in the emotion measurement methods that need to be improved: First, there are fewer studies that measure tourists’ emotions based on their psychological and physiological reactions, and most still use tracking surveys and questionnaires, which cannot accurately and instantly measure the emotions of museum tourists. Second, there are fewer studies on psychological and physiological reaction-based emotion measurement for museum tourism research, and there is a problem with capturing the physiological emotional states that occur in an instant. Third, the results of existing research on psychological and physiological reaction-based emotion measurement have good immediacy and accuracy, but the experimental environment requirements are strict, which does not meet the actual environment of museum tourists, and there are fewer studies conducted in the museum tourism field. This provides a research idea for this article to comprehensively measure the real-time digital emotional experience of museum tourists based on their psychological and physiological reactions from the perspective of tourists’ psychology.

## 3. Experimental design

### 3.1. Experimental time and location

The experimental time is chosen to be in July–August 2022. This period is the hottest month in 2022, with hot weather and intense heat, which easily leads to irritability and lack of concentration. If the experimental results are valid in the relatively unfavorable conditions of July–August, the effects in other months would be even more effective. The experimental location is chosen to be the screening room of Tianyi Pavilion Museum (5A) in Ningbo. Tianyi Pavilion Museum is a thematic museum with a core focus on book culture, and it serves as a typical research subject. In front of the exit of Tianyi Pavilion, there is a dedicated digital technology application environment - the “Tianyi Pavilion Documentary” screening room, which specifically plays a video showcasing the establishment, prosperity, and decline of Tianyi Pavilion. The video mainly evokes a sense of sadness and has a total duration of approximately 15 min.

### 3.2. Experimental subjects

Translation: According to the requirements of psychological experiments, a sample size of more than 30 subjects is considered a large sample. In this experiment, a convenience sampling method was used, and a total of 42 tourists were recruited. The experimental subjects were primarily female, accounting for 73.8%, while males accounted for 26.2%. The age range was between 15 and 50 years old, with the main concentration in the age groups of 15–18, 26–35, and 36–45, accounting for 28.6, 23.8, and 23.8%, respectively. In terms of education, the majority had a master’s degree or above, followed by junior high school or below, and then a bachelor’s degree, accounting for 31, 19, and 19%, respectively. Associate degrees accounted for 16.7%, and high school accounted for 14.3%. In terms of occupation, students accounted for the majority, reaching 40.5%, followed by personnel engaged in public institutions, accounting for 21.4%, with company employees and freelancers both accounting for 11.9%, and others were negligible. In terms of income distribution, the majority had an income below 1,000 yuan, accounting for 38.1%, followed by an income above 10,000 yuan, accounting for 35.7%, with 5,000 to 10,000 yuan coming next, accounting for 14.3%, and finally, 1,000 to 5,000 yuan, accounting for 11.9%.

### 3.3. Experimental instruments

The tools used to collect tourists’ brainwave signals in this experiment include the BrainLink EEG device (Professional Research Edition) manufactured by Neurosky, a laptop, a mobile phone, and a compatible EEG biofeedback system. The BrainLink (Professional Research Edition) EEG device primarily processes brainwave signals through data input, filtering, electrode positioning, segmentation, and ICA analysis (Independent Component Analysis) to eliminate artifacts. The preprocessed data is then subjected to short-time Fourier transform for the extraction of energy from the brainwave signals.

**Figure 1:**
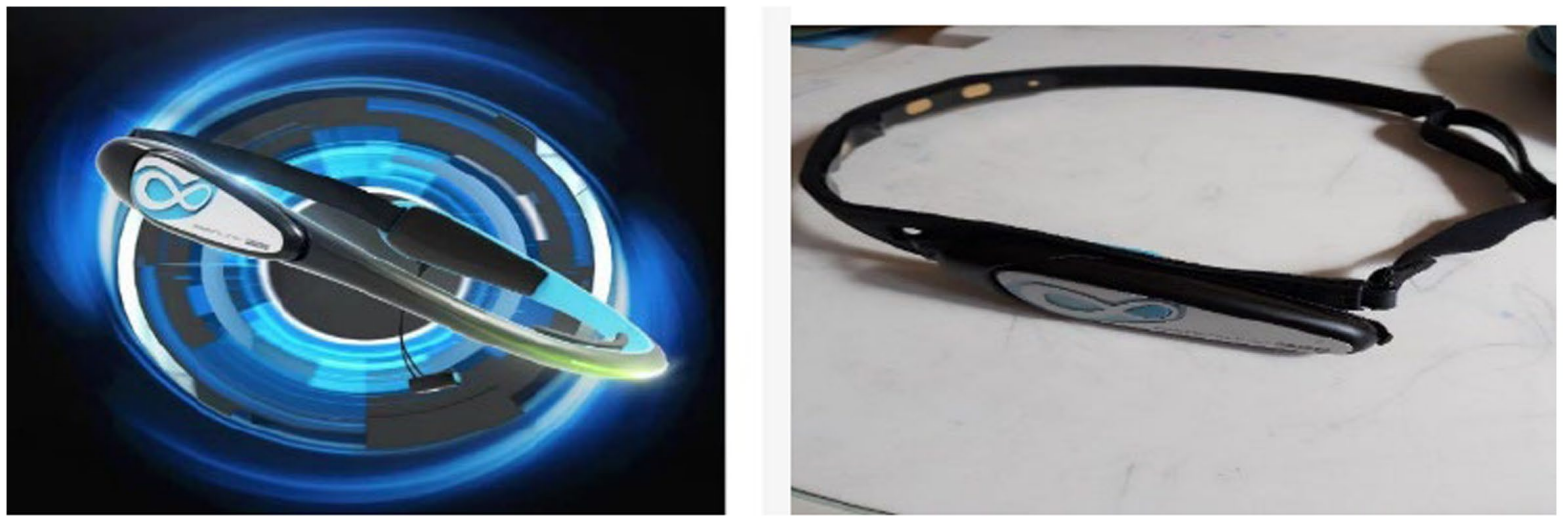
BrainLink (professional research edition).

The BrainLink EEG device (Professional Research Edition) can directly obtain the energy levels of delta waves, theta waves, alpha waves, beta waves, and gamma waves in real-time after signal processing, as shown in Figure 1. Currently, the effectiveness of the BrainLink device in collecting EEG signals has been verified by various research teams both domestically and internationally.

### 3.4. Experimental procedures

specific experimental steps: (1) Prior to the experiment, provide a brief introduction of the process and precautions to tourists in order to ensure smooth completion without interference such as phone calls or leaving midway, and obtain their consent. (2) After obtaining consent, monitor tourists’ brainwaves throughout the screening of the documentary until its conclusion, followed by completion of “Museum Tourism PAD Emotional Scale” questionnaire. (3) Collect data indicators such as tourists’ brainwaves, skin temperature, ambient temperature, and time throughout the experiment. (4) After completing the questionnaire, conduct in-depth interviews with tourists to gather qualitative data that aims to further comprehend their genuine emotions and experiences after viewing the video while also providing more comprehensive details and supplementary explanations for quantitative test results.

### 3.5. Experimental indicators and introduction

Tourist PAD emotional values: Consists of three parts, namely P (pleasure), A (arousal), and D (dominance). P: pleasure, reflects the degree of pleasure that is evoked in the inner emotions of the tourists after watching the documentary. A: arousal, reflects the level of neural physiological activation and excitement of the tourists after watching the documentary. D: dominance, reflects the degree of control that the tourists have over the situation and others in their inner emotions after watching the documentary.

Magnitude of ∂,β,γ,δ,θ energies: given the computational and practical nature of the data, we have adopted Guo et al. (2019) approach to select the mean values of different types of brainwave energies during the monitoring period for experimental subjects.

## 4. Experimental results and analysis

### 4.1. Psychological response emotion measurement: assessing the affective states of museum visitors in a digital technology application context using the PAD emotional model

By utilizing formula (1), the affective states of museum visitors’ PAD were computed within the digital technology application environment and presented in Table 2. In the context of digital technology applications, the mean *p*-value of museum visitors is 0.055 with a standard deviation of 0.783 and a median of 0.25, indicating an overall positive emotional state among visitors. Visitors with *p*-values >0 account for 54.8% of the total, while those with *p*-values = 0 and < 0 account for 16.7 and 28.5%, respectively, suggesting that approximately half (54.5%) are in a positive emotional state and nearly one-third (28.5%) are experiencing negative emotions. The mean A-value of museum visitors is −0.048, with a standard deviation of 1.302 and a median of −0.25, indicating that visitors are generally in a low state of arousal. Visitors with A-values >0 account for 42.8% of the total, visitors with A-values = 0 account for 4.8%, and visitors with A-values <0 account for 52.4%. This suggests that approximately half (52.4%) of the visitors are experiencing low levels of excitement while almost half (42.8%) are highly aroused.

**Table 2:** The affective values and emotional tendencies of museum-goers’ PAD within the digital technology application context.

Sample number	P	A	D	Emotional state	Emotional tendency	Sample number	P	A	D	Emotional state	Emotional tendency
1	0.75	−1.75	0.5	Gentleness	Negative	22	0	0.25	0	Anxiety	Negative
2	0.5	−0.5	0.25	Gentleness	Negative	23	0.5	1	−0.5	Fear	Negative
3	0	−0.5	−0.5	Boredom	Negative	24	0.25	−1	−0.25	Boredom	Negative
4	0.25	−0.25	0.75	Gentleness	Negative	25	0.5	−0.75	−1.5	Dependence	Positive
5	−1.25	−1.75	1.25	Boredom	Negative	26	0	0	0	Anxiety	Negative
6	0.75	0.75	0.5	Surprise	Positive	27	0	−0.75	−0.75	Boredom	Negative
7	−0.25	−1.25	−0.25	Boredom	Negative	28	−1	−1.5	0.5	Boredom	Negative
8	0.5	1.5	1.5	Surprise	Positive	29	1.25	1.75	0.5	Surprise	Positive
9	0.5	−2	1	Gentleness	Negative	30	0.5	0.5	1.25	Gentleness	Negative
10	0	−0.75	0.75	Gentleness	Negative	31	1.5	−1	1.25	Relaxation	Positive
11	−0.25	0.5	0.25	Anxiety	Negative	32	−0.25	−0.25	0.5	Anxiety	Negative
12	1	0.25	−0.5	Gentleness	Negative	33	0.57	−0.75	−0.75	Dependence	Positive
13	−1.5	1	0.5	Resentment	Negative	34	1.25	−2.5	−2.75	Dependence	Positive
14	0.5	1	−0.25	Surprise	Positive	35	−0.75	1.5	2	Contempt	Negative
15	−0.75	−1	−1	Boredom	Negative	36	0	1.5	1.75	Joy	Positive
16	−2.25	1	−2	Resentment	Negative	37	0.25	−1.25	−0.5	Boredom	Negative
17	−0.5	−0.25	0	Sadness	Negative	38	0.25	1.5	1.5	Surprise	Positive
18	0.25	−2.50	−2.25	Dependence	Positive	39	0.5	2.25	−1.75	Fear	Negative
19	−1.75	−1.5	0	Boredom	Negative	40	0.25	2	1	Surprise	Positive
20	0	−0.25	0	Anxiety	Negative	41	0.25	2	1	Surprise	Positive
21	−0.5	0	−0.25	Sadness	Negative	42	0.5	1.75	2	Joy	Positive

The mean D-value of museum visitors is 0.113, with a standard deviation of 1.113 and a median of 0.125, indicating that visitors are generally in an active control state and exhibit strong interaction with their surrounding environment. Visitors with D-values greater than zero account for half the total population, while those with values equal to zero make up 11.9%, and those below zero comprise 38.1%. This suggests that half of all visitors are actively engaged in controlling their experience, while just over one-third remain passively receptive. It is noteworthy that visitors with mild emotions tend to exhibit negative emotional tendencies, as indicated by Table 2 and visitor feedback from interviews. This can be attributed to two factors: Firstly, the theme of materials presented in digital technology applications often relates to sadness. The current playback theme predominantly features melancholic elements and focuses on the entire process of Tianyi Pavilion’s establishment, prosperity, and decline. Additionally, two-thirds of video time is dedicated to depicting the decline of Tianyi Pavilion. Secondly, the presence of negative emotional states among visitors does not necessarily indicate a poor emotional experience. In fact, the sublimation of these negative emotions can effectively enhance the quality of visitors’ tourism experience. For instance, a thematically designed emotional encounter centered around the color black may elicit negative emotions in visitors. However, following their participation in a guided museum tour, their comprehension of associated events deepens, leading to an internal sublimation of their initial negative emotional state and ultimately enhancing their overall emotional experience (Li and Liu, 2021; Wang and Li, 2021).

### 4.2. Physiological response emotion measurement: measuring museum visitors’ emotions in a digital technology application environment through brainwave signal analysis

**Figure 2:**
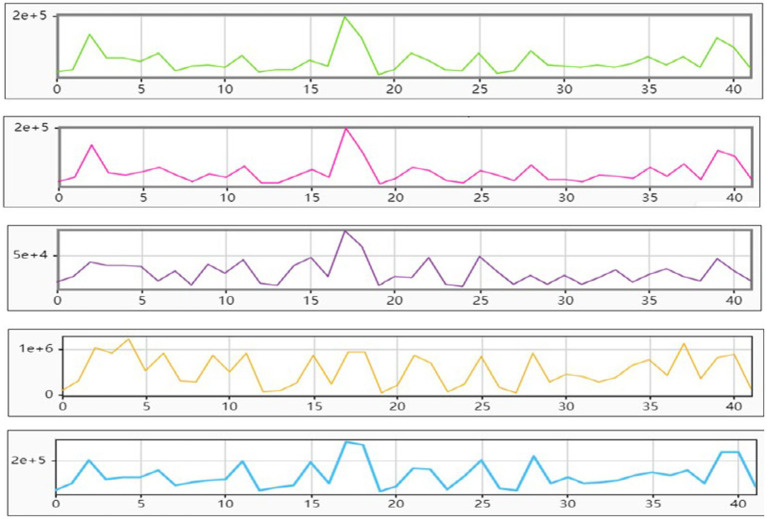
The energy levels of the waves are arranged in the following order: δ, β, γ, δ, θ.

Spectrum analysis of brainwave signals from museum visitors’ neural activity. By utilizing electroencephalography (EEG) to acquire and preprocess brainwave signals from museum visitors, the energy of δ, β, γ, δ, and θ waves in a quasi-experimental setting was extracted as depicted in Figure 2. According to Figure 2, the mean energy of δ waves is 54.87 (SD = 38.5, median = 41.88), β waves is 53.94 (SD = 37.45, median = 43.37), and γ waves is 26.83 (SD =16.76, median = 22.73). Additionally, the mean energy of θ waves is found to be at a lower level with a value of only11 0.65(SD = 7 0.5)

Refining the recognition and classification of museum visitors’ emotions through Support Vector Machine. Using Python to identify and classify the emotions of museum visitors involves several steps. Firstly, emotional tendencies are classified as positive or negative based on the PAD emotion values of visitors. Next, principal component analysis is performed on the EEG signal energy data of these visitors, followed by random selection of 70% samples for training and 30% for testing purposes. Finally, linear SVM, polynomial kernel SVM, Gaussian kernel SVM and Sigmoid SVM are utilized to predict their emotional states. Separate predictions were made for museum visitors’ positive or negative emotional tendencies during physiological measurements, considering the potential deviation from their true inner emotions when they are in a moderate state.

According to Table 3, the emotional tendencies of museum visitors were classified as negative in both quasi-experimental and digital technology application environments with a 73% success rate for emotional classification prediction using linear SVM. The emotional classification prediction results using polynomial kernel SVM are as follows: the training set achieved a success rate of 74.2%, and the test set achieved a success rate of 72.7%. Meanwhile, the emotional classification prediction results using Gaussian kernel SVM showed that the training set had a perfect score of 100%, but only reached a success rate of 72.7% on the test set. Unfortunately, Sigmoid SVM failed to predict museum visitors’ emotional tendencies in this quasi-experimental environment. Therefore, in the quasi-experimental environment, linear SVM, polynomial kernel SVM, and Gaussian kernel SVM can all identify and classify the emotional tendencies of museum visitors, with success rates of over 70%.

**Table 3:** Test results of different support vector machine algorithms for classifying the emotional tendencies of museum visitors.

	Success rate of predicting positive category when classified as mild		Success rate of predicting negative category when classified as mild	
Method	Training dataset	Testing dataset	Training dataset	Testing dataset
Linear SVM	–		0.73	
polynomial kernel SVM	0.935	0.636	0.742	0.727
Gaussian kernel SVM	0.903	0.535	1	0.727
Sigmoid SVM	0.516	0.545	–	–

— represents unstable results and classification failure.

When classifying museum visitors as positive in the quasi-experimental environment of digital technology application, only polynomial kernel SVM is capable of successful prediction with a test success rate of 63.6%, which is significantly lower than the success rate achieved when classifying museum visitors as negative under the same conditions. Linear SVM, Gaussian kernel SVM, and Sigmoid SVM exhibit unsatisfactory performance in predicting and categorizing the emotions of museum visitors. This aligns with the findings from interviews conducted with museum visitors, which suggest that individuals experiencing mild emotions tend to have a negative emotional inclination.

In summary, the utilization of EEG signals and the PAD emotion model for real-time digital measurement of tourists’ emotional experiences in a quasi-experimental environment is both feasible and reliable. In such an environment, it is necessary to integrate visitors’ psychological and physiological reactions to accurately measure their emotions in real-time, resulting in valuable outcomes. The emotional inclinations of museum visitors are relatively mild and predominantly negative, which is consistent with the feedback and brainwave signal recognition findings from museum visitors.

## 5. Emotional traits of museum visitors in the vontext of digital technology application

Analysis of the emotional tendencies of museum visitors under digital technology: In a quasi-experimental setting, the overall emotional tendency of museum visitors in an environment with digital technology application is predominantly negative, accounting for 66.7%. This outcome is closely linked to the themes of presented digital content. In fact, this is in line with the findings from the interviews: out of all the experimental subjects interviewed, only two tourists expressed skepticism towards the documentary’s ability to enhance their travel experience, while all other participants deemed it necessary and impactful.

Among them, Museum visitors with positive emotional tendencies exhibit the following characteristics: they are predominantly female, comprising 79% of the total; their ages are primarily concentrated in the age groups of 26–35, 15–18, and above 46 years old; their educational background is mainly at the college and master’s degree level or higher; their occupations are mostly students, while their income is distributed mainly below 1,000 yuan, between 5,000–10,000 yuan, and above 10,000 yuan. Their preferred viewing position is primarily located at position eight.

Characteristics of negative emotional tendencies among museum visitors: predominantly female, accounting for 71%; age mainly concentrated in 15–18 years old and 36–45 years old; education level mainly consists of master’s degree, undergraduate, and junior high school and below; occupation mainly students and public service institutions; income mainly distributed below 1,000 yuan and above 10,000 yuan; viewing position mainly position 5, position 7, and position 8. It can be seen that regardless of positive or negative emotional tendencies among museum visitors, they are consistent with the overall distribution of museum visitors.

According to Table 4, the emotional characteristics of museum visitors with different demographic features are as follows: (1) In terms of age, while the majority of museum visitors fall within the 15–18 age range, those aged between 26 and 35 exhibit a higher propensity towards positive emotions without significant numerical advantage. This suggests that individuals in this age group may be more predisposed to experiencing positive emotions during their visit compared to other age groups. (2) In terms of education, the top three categories with the highest number of visitors are: Master’s degree or above, Bachelor’s degree, and Junior high school or below tied for third place. However, when it comes to positive emotions, those with a Master’s degree or above and an Associate degree are tied for first place while other educational levels come in second. The ranking of negative emotions aligns with the total number of visitors. This suggests that museum-goers holding advanced degrees tend to experience more positive emotions. (3) In terms of occupation, government employees rank second in total museum visitors, but exhibit the lowest frequency of positive emotions. The ranking for negative emotions corresponds with the overall number of visitors. This suggests that compared to other occupations among museum-goers, government employees display a lower propensity towards positive emotions. (4) In terms of income, museum visitors with a monthly income ranging from 5,000 to 10,000 yuan are the only group where the number of visitors exhibiting a higher propensity towards positive emotions surpasses those displaying a tendency towards negative emotions. (5) In terms of viewing position, the inclination towards positive and negative emotions among museum visitors corresponds with the overall visitor count. (6) In terms of travel mode, while the inclination towards positive and negative emotions among museum visitors corresponds with the total number of visitors, only those who visit museums with friends exhibit a greater tendency towards positive emotions than negative ones.

**Table 4:** Emotional traits of museum attendees based on demographic characteristics.

	Gender	Age	Education level	Occupation	Income	Viewing position	Mode of travel
General	Female	15–18 years old	Master’s degree and above	Student	Below 1,000 yuan	Position 8	Family travel
Positive	Female	26–35 years old	Associate degree master’s degree and above	Student	Below 1,000 yuan	Position 8	Family travel
negative	Female	15–18 years old	Master’s degree and above	Student	Below 1,000 yuan	Position 5, 7, 8	Family travel

## 6. Conclusion and prospects

### 6.1. Conclusion

In the context of digital technology application, we utilize EEG signals and the PAD emotional model to comprehensively measure museum visitors’ immediate digital emotional experience based on their physiological and psychological reactions from a tourist psychology perspective. The results indicate that: (1) In a quasi-experimental setting, linear SVM, polynomial kernel SVM, and Gaussian kernel SVM demonstrate the ability to accurately detect and classify the emotional tendencies of museum visitors with success rates of 73, 72.4, and 72.4%, respectively. (2) In a quasi-experimental setting, it is feasible to employ EEG signals and the PAD emotional model for assessing the immediate digital emotional experience of museum visitors, with reliable outcomes.

Secondly, the analysis of museum visitors’ emotional characteristics based on digital emotional experience measurement reveals that: (1) Visitors tend to exhibit negative emotions in response to the theme of the digital materials presented. (2) Regardless of their emotional tendencies, whether positive or negative, female visitors are the predominant demographic with an age range of 15–18 years old. The majority of these visitors are students with incomes ranging from below 1,000 yuan to above 10,000 yuan. They tend to occupy position 8 and often travel with their families. This distribution is consistent with the overall statistics of museum visitors. (3) The analysis of emotional characteristics among museum visitors from diverse demographic groups reveals that individuals aged 26–35 exhibit a greater propensity towards positive emotions in comparison to other age cohorts; visitors holding a college degree exhibit a greater propensity for positive emotions in comparison to those with alternative educational backgrounds; visitors from government institutions exhibit the lowest propensity for positive emotions compared to visitors from other professions; among the various categories of museum visitors, only those who spend between 5,000–10,000 yuan and travel with friends exhibit a greater number of positive emotions than negative ones.

### 6.2. Prospects

The research on measuring the emotions of museum visitors is still in its preliminary stage, and this article has certain limitations. Future studies can be conducted to address the following areas: (1) Although a sample size of 42 people meets the requirements for large samples in psychology, a larger sample size would provide more distinct characteristics in analyzing and summarizing emotional tendencies among museum visitors. In the future, it is advisable to further increase the sample size. (2) This experiment employed videos with a melancholic theme to synthesize the emotional inclinations of museum visitors in a digital technology application environment, which tended towards negative emotions. In future studies, it is recommended to further investigate the emotional tendencies of museum visitors in digital technology application environments featuring themes of joy and neutrality. (3) When measuring the emotional responses of museum visitors through psychophysiological measurements, brainwave energy magnitude is the primary characteristic utilized for selection. However, it is recommended that other brainwave signals such as PSD be explored for research purposes in the future. We primarily employ support vector machines as the research methodology for emotional recognition of brainwave data. In the future, we may explore other approaches such as convolutional neural networks to further enhance the accuracy of emotional recognition.

## Data availability statement

The original contributions presented in the study are included in the article/supplementary material, further inquiries can be directed to the corresponding author.

## Author contributions

ZQ supervised the article, while TZ independently conducted the research design and writing. TZ and WG conducted the data collection and analysis. CZ and DJ done the literature review and objective correction. TZ, ZQ, WG, CZ, and DJ contributions to the revision and further improvement of the article, provided, constructive suggestions and recommendations to ensure accurate expression of the complex research findings. All authors contributed to the article and approved the submitted version.

## Conflict of interest

WG was employed by China Yangtze Power Co., Ltd.

The remaining authors declare that the research was conducted in the absence of any commercial or financial relationships that could be construed as a potential conflict of interest.

## Publisher’s note

All claims expressed in this article are solely those of the authors and do not necessarily represent those of their affiliated organizations, or those of the publisher, the editors and the reviewers. Any product that may be evaluated in this article, or claim that may be made by its manufacturer, is not guaranteed or endorsed by the publisher.

## Footnotes

^1^Emotional state represents the interplay between individuals and their social needs, manifesting in diverse contexts such as elation, indignation, grief, and contentment. It can manifest as affective states such as mood, passion, and stress.

^2^Emotional inclination refers to an individual’s subjective inner preference and evaluation towards a specific object.
